# Attachment styles and happiness in the elderly: the mediating role of reminiscence styles

**DOI:** 10.1186/s12877-022-03053-z

**Published:** 2022-04-20

**Authors:** Khodamorad Momeni, Rozita Amani, Parisa Janjani, Mohammad Reza Majzoobi, Simon Forstmeier, Parisa Nosrati

**Affiliations:** 1 Department of Psychology, Faculty of Social Sciences, Razi University, Kermanshah, Iran; 2 Department of Psychology, Faculty of Economics & Social Sciences, Bu-Ali Sina University, Hamedan, Iran; 3 Cardiovascular Research Center, Health Institute, Kermanshah University of Medical Sciences, Kermanshah, Iran; 4 Developmental Psychology and Clinical Psychology of the Lifespan, University of Siegen, Siegen, Germany

**Keywords:** Attachment styles, Reminiscence styles, Happiness, Aged, Iran

## Abstract

**Background:**

The current study aims to investigate the relationship between attachment styles and happiness through the mediating role of reminiscence styles in the elderly.

**Methods:**

This was a correlational study of structural equations modelling (SEM) type. The statistical population included all the elderly aged at least 60 years living in Kermanshah province, Iran in 2021, among whom 380 (182 men and 198 women) were selected using convenience sampling method. Participants filled out the questionnaires of Adult Attachment Styles, Oxford Happiness, and Amani et al.’s Reminiscence Styles.

**Results:**

The results indicated that secure attachment style has a positive and negative relationship with positive reminiscence (PR) and negative reminiscence (NR), respectively. However, the opposite held true for both avoidant and ambivalent attachment styles. It was also found that secure attachment style has a positive relationship, and avoidant and ambivalent attachment styles have a negative relationship with happiness. Moreover, participants’ gender and age had no moderating effect on the mentioned relationships. The results of SEM indicated that secure and ambivalent attachment styles were associated with happiness through both PR and NR, and avoidant attachment style was associated with happiness only through NR.

**Conclusions:**

The findings emphasize the significance of the development of internal working models based on the kind of parent-child’s reminiscences and narratives, and the lifelong effects of these models.

## Background

In 2010, the World Health Organization estimated that the papulation of the elderly will cover about 16% of the global population by 2050, and the growth rate seems to be more in developing countries [1]. Iran, as one of these developing countries, is no exception to this population change. According to the Statistical Center of Iran, the elderly population of this country has increased from 6.4% in 1966 to 9.1% in 2016 [2]. One of the consequences of aging is a decrease in the extent of happiness [3]. Studies have shown that more than ninety percent of the elderly have a moderate level of happiness [4].

Happiness as a positive inner experience seems to be one of the indicators of mental health that stem from the cognitive and emotional evaluation people carry out in their own lives [5]. Studies have demonstrated that happiness is associated positively with prominent variables in the elderly such as life satisfaction [6, 7], mental health [7–9], and social health [10]. Studies have introduced the role of various variables such as economic status [4, 11], social participation [12], self-esteem [13, 14] and perceived social support [15] in predicting happiness in the elderly. Attachment style, as well, has been presented as one of the most central predictors of happiness [16, 17].

Attachment is defined by Hollist and Miller [18] as a deep emotional connection and affective communication with certain people in life whom a person feels enjoyment and comfort when interacting with. Shaver and Hazan [19] divided adult attachment styles into three categories of secure, avoidant, and ambivalent. Secure people, who have a history of warm and responsive interaction with their attachment figures (parents), are usually characterized by having a positive view of themselves and others [20]. Avoidant individuals, however, are usually uncomfortable having close relationship with others and they are likely to find it hard to trust others. Finally, ambivalent adults, who seems to be overly dependent on others, believe that others are reluctant to build a close relationship with them [21]. According to the attachment theory, attachment style appears to have lifelong effects, and determine how individuals cope with their interpersonal problems during their lives [13, 22–24]. Therefore, secure attachment is thought to be a protective resource for the elderly as well [25].

Although the aforementioned studies have outlined the relationship between attachment styles and happiness in the elderly, few studies have examined how this relationship may work, namely the pathways through which attachment styles may affect happiness. Almost no studies to our knowledge have examined this pathway in the elderly. For instance, Kamari and Shekhaleslami [26] stated that attachment styles are associated with happiness through optimism in college students. Given that according to Butler’s theory [27], one of the determining factors in the life of the elderly is how they review the memories they had during their lives (referred to as reminiscence styles), this variable, whose prominent role in the life of the elderly is well determined [27], may be a reasonable mediator for the relationship of attachment styles and happiness. However, no study, as of yet, has focused on reminiscence styles as the mediator variable between attachment styles and happiness neither in ages other than the elderly nor in the elderly.

Reminiscence refers to the process of thinking or talking about past experiences and memories [28]. Watt and Wong [29] divided reminiscence into six types including integrative (reviewing life and finding meaning and value from past experiences), instrumental (reminiscing about past experiences to solve problems and reinforce present performance), transmissive (recalling memories to share specific knowledge), escapist (reviewing the past and perceiving it as a better time than it is now), obsessive (reminiscing about negative times in life and repeatedly thinking about them), and narrative reminiscence (reviewing the past experiences in the shape of a story). Studies have illustrated the impact of integrative reminiscence on depression, integrity, self-esteem and life satisfaction [30], instrumental reminiscence on coping [31] and depression [32, 33], transmissive reminiscence on general health [34], and narrative reminiscence on happiness [35] and the meaning of life [36] in the elderly. Studies have also shown that attachment styles play a significant role in shaping the type of reminiscence in individuals. For example, Molinari et al. [37] found that compared to unsecure older adults, secure ones scored higher on the teach/inform (transmissive) reminiscence. They also found that there is a significant negative correlation between fearful attachment and teach/inform reminiscence.

Although studies have shown the effectiveness of reminiscence-based interventions on increasing happiness in the elderly [38–41], fewer studies have examined the relationship between reminiscence styles, as a self-report variable, and happiness in the elderly. Webster [16] indicated that on the one hand, attachment style is a significant predictor of four types of reminiscence, namely bitterness revival (obsessive), identity (integrative), problem solving (instrumental) and teach/inform (transmissive), and on the other hand, bitterness revival, boredom reduction, identity, and problem-solving reminiscences had a significant negative relationship with happiness. Besides, conversation and teach/inform reminiscences showed a significant positive relationship with happiness. Although Webster’s study has somewhat outlined the relationship among attachment styles, reminiscence styles and happiness, no information is provided on how all three of these variables are related together in the form of a structural model. Therefore, the study of the relationship between these three variables in the form of a structural model can bridge one of the existent gaps in the research literature related to this field of inquiry, particularly in the elderly age group, wherein the reminiscence construct serves a very decisive role.

There are also contradictory findings regarding the role of participants’ gender and age in the extent and type of reminiscence in the elderly. For example, Webster [16] did not find out any gender difference in the extent and type of reminiscence in the elderly. Webster also found that older people are more inclined to do death preparation, intimacy maintenance, and teach/inform reminiscences, and that young people more tend to do bitterness revival, identity, and problem-solving reminiscences. However, Webster and McCall [41] demonstrated that women score higher on identity reminiscence and lower on bitterness revival. Webster and McCall also figured out that younger people score higher on boredom reduction, bitterness revival and identity, and older people were more likely to do teach/inform and death preparation reminiscences.

**Fig. 1:**
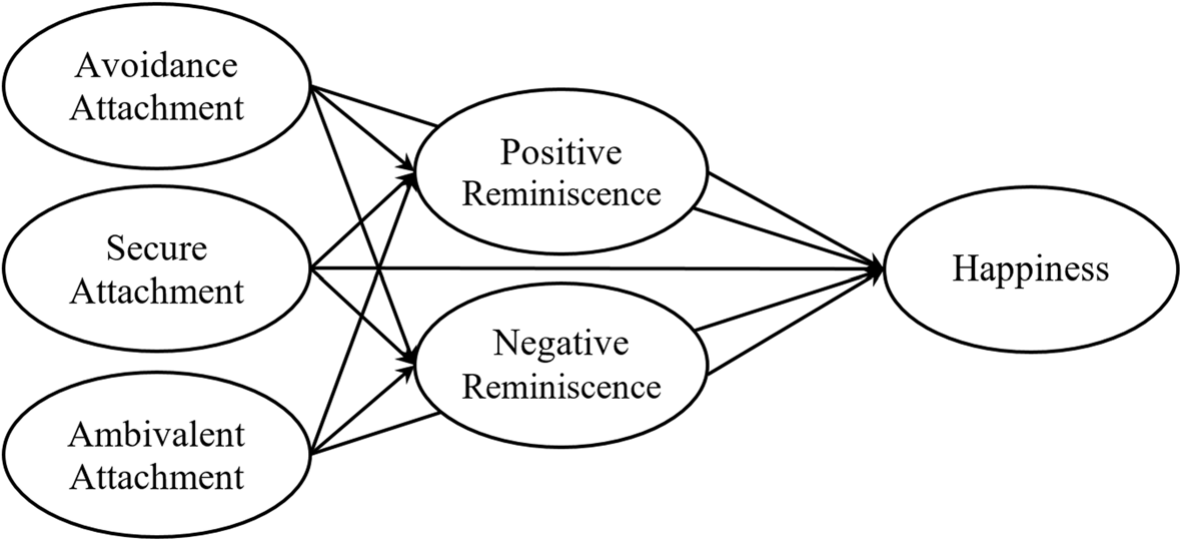
Hypothesized model for the relationship between attachment styles and happiness through mediating role of reminiscence styles

Given the growing population of the elderly in the world, of which Iran is no exception, and also the need to better understand the important variables of the elderly and how these variables are related to each other, conducting studies that show the relationship between important variables of the elderly are thought to be crucial. Therefore, this study was conducted to determine how two of the essential psychological variables like attachment style and happiness are related to each other in the elderly. The implementation of such studies makes the mediating variables in the relationship between the mentioned essential variables known, based on which the specialists in the field of clinical psychology can prepare valuable therapeutic protocols to increase the happiness of the elderly. Therefore, considering the gaps and contradictions in the research literature related to this field of inquiry, this study was to investigate the relationship between attachment styles and happiness through the mediating role of reminiscence styles. The hypotheses of this study were as follows: (1) avoidant attachment style is negatively related to positive reminiscence style, which in turn is positively related to happiness (2) avoidant attachment style is positively related to negative reminiscence style, which in turn is negatively related to happiness (3) secure attachment style is positively related to positive reminiscence style, which in turn is positively related to happiness (4) secure attachment style is negatively related to negative reminiscence style, which in turn is negatively related to happiness (5) ambivalent attachment style is negatively related to positive reminiscence style, which in turn is positively related to happiness (6) ambivalent attachment style is positively related to negative reminiscence style, which in turn is negatively related to happiness, (7) participants’ gender and age moderate the relationship between attachment styles and reminiscence styles, and (8) participants’ gender and age moderate the relationship between reminiscence styles and happiness. The hypothesized model is presented in Fig. 1.

## Method

### Study design and participants

The method of the present study was correlational of SEM type. The statistical population included all the elderly aged at least 60 years living in Kermanshah province, Iran in 2021, among whom 380 (182 men and 198 women) were selected as the study sample using convenience sampling method. Regarding the sample size, it is worth noting that Stevens as cited by Hooman [42] stated that considering 15 items for each predictor variable in the multiple regression analysis with the ordinary least squares, of the minimum squared standard is a good rule of thumb. Based on this, it can be stated that because SEM in some respects is completely related to multivariate regression, 15 cases for each variable measured in SEM are not irrational [42]. Loehlin as cited by Hooman [42] states that for models with two or four factors, the researcher must plan to collect data from at least 100 participants. Therefore, the sample size of this study seems sufficient to perform a SEM. Inclusion criteria were (1) age over 60 years, (2) willingness to participate in the study, (3) lack of experience of grief of losing loved ones in the last six months, and (4) absence of any severe mental disorders based on participants’ medical records (to check this criterion, we asked participants if their medical records had a history of diseases such as Alzheimer's, dementia and suchlike.).

### Measures

#### Attachment Styles Questionnaire (ASQ)

ASQ was developed by Dost Mohammadi [43] based on items of Hazan and Shaver’s [44] adult attachment scale (AAS). This questionnaire is comprised of 15 items that measure three attachment styles of secure, avoidant, and ambivalent. Participants answer questions through a five-point Likert scale ranging from one (strongly disagree) to five (strongly agree). There are five items for each attachment style and the minimum and maximum scores for each style are from five to 25. Dost Mohammadi [43] reported the reliability coefficient for ASQ to be 0.81 through the retest method and 0.87 through Cronbach’s alpha method. Using Cronbach’s alpha method in the current study, we obtained the Cronbach’s alpha of 0.67, 0.65, and 0.61 for avoidant, secure, and ambivalent components, respectively.

#### Oxford Happiness Questionnaire (OHQ)

OHQ, developed by Argyle et al. [45], has 29 items, each of which has four options ranging from zero to three. The sum of the scores of the 29 items determine the total score of the scale. People get a score between zero to 87, with higher scores indicating higher happiness. The questionnaire includes five components of life satisfaction, self-esteem, subjective well-being, self-satisfaction, and positive mood. Using Cronbach’s alpha method, Argyle et al. [45] reported the validity of this questionnaire to be 0.9. They also confirmed the divergent validity of OHQ with Beck and Clark [46] depression inventory and reported its reliability through Cronbach’s alpha method to be 0.90. Alipoor and Noorbala [47] as well figured out that the validity of OHQ on the Iranian sample was 0.93. Cronbach’s alpha for the total score of OHQ was 0.94 in the current study.

#### Elderly Reminiscence Questionnaire (ERQ)

ERQ was developed and validated by Amani et al. [48] based on Watt and Wong [29] questionnaire to measure the extent and styles of reminiscence in Iranian elderly. This questionnaire is comprised of 30 items that measure the five subscales of narrative-transmissive, obsessive, integrative, death preparation, and escapist reminiscence. Each item is scored on a 5-point Likert scale, ranging from “never” to “very much”. The minimum and maximum scores are zero and 120, respectively, with higher score indicating higher level of reminiscence, and vice versa. The sum of the scores of narrative-transmission and integrative subscales provides the positive reminiscence (PR) score and relatedly, the sum of the scores of obsessive, death preparation, and escapist subscales provides the negative reminiscence (NR) score. Using Cronbach’s alpha method, Amani et al. [48] figured out that the internal reliability of ERQ is 0.904 for all subscales and are 0.904, 0.826, 0.795, 0.812, and 0.774 for narrative-transmissive, obsessive, integrative, death preparation, and escapist subscales, respectively. In the current study, the internal reliability of ERQ through Cronbach’s alpha method was 0.904 for all items, and were 0.808, 0.881, 0.858, 0.799 and 0.844 for narrative-transmissive, obsessive, integrative, death preparation, and escapist subscales, respectively.

### Procedure

Having obtained the necessary permissions to conduct the research from the ethics committee, we conducted the preliminary stage of the research. Due to the concurrence of the current study and the peak stage of COVID-19 pandemic and cross-country lockdown in Iran, we could not find the elderly in parks, cultural and entertainment centers, day care centers and mosques in person. Therefore, we designed the aforementioned questionnaires in the form of online ones and recruited students who were studying general psychology at Razi University and also living in Kermanshah city or other cities of Kermanshah province to send the questionnaires to the elderly they knew in their neighbors and relatives who were eligible with respect to the inclusion criteria of the study. These students were passing the “Assessment and Measurement in Psychology” module and this task were proposed to them as the practical part of this module. Of course, they were free to accept this task. To this end, we sent an email to the volunteer students (*n*=42) containing the web link of online questionnaires as well as elaborative explanation concerning research objectives, inclusion criteria, and the way to implement questionnaires in order to guide them on how to conduct data gathering stage. Meanwhile, we asked students not to visit the elderly in person to avoid the risk of transmitting COVID-19 virus to them. Therefore, the students informed the elderly around them by phone, and the web link of the questionnaires was provided to the elderly through an email or other social networks. Before the elderly reached the stage of filling out the questionnaires, the students presented the research objectives to them, provided them with necessary explanations regarding the lack of identity information on questionnaires, the participants’ privacy and confidentiality of their personal information, and finally obtained their informed consent for participating in the study. Participants were also asked to answer preliminary questions concerning inclusion criteria, and those who met the inclusion criteria were allowed access to the main questionnaires. It should be noted that if participants did not have a smartphone or tablet, or were not able to answer questionnaires due to old age and lack of skills, their children or spouse living with them were asked to help them with this task. This help was usually provided by reading questions to them and entering their answers into the questionnaires. One of the advantages of conducting this study online was that the questionnaires were designed in such a way that they would not be sent until they were fully answered. Therefore, there was no missing data. Finally, reviewing 400 questionnaires completed by the participants, we discarded 20 distorted questionnaires (for reasons such as age outside the scope of this study, and suffering from debilitating mental and physical illnesses) and analyzed 380 questionnaires as the final sample of the research. The data obtained from the questionnaires were analyzed using Pearson’s correlation coefficient, hierarchal linear regression, and SEM in SPSS-21 and LISREL-9.1 software.

## Results

Table 1 indicated the sociodemographic characteristics of the participants.

**Table 1:** Sociodemographic Characteristics of the Participants

characteristics	Group	Number (percent)
Age	60-69	260 (68.4)
	70-79	88 (23.2)
	80-89	27 (7.1)
	90-99	5 (1.3)
Sex	Men	182 (47.9)
	Women	198 (52.1)
Marital status	Married	294 (77.37)
	Single (Death of spouse)	78 (20.53)
	Single (divorce)	4 (1.05)
	Single (not getting married)	4 (1.05)
Education	High school degree and lower	191 (50.3)
	Diploma degree	86 (22.6)
	Associate’s degree	31 (8.2)
	Bachelor’s degree	48 (12.6)
	Master’s degree	19 (5)
	Doctorate degree	5 (1.3)
Children	With children	368 (96.8)
	Without children	12 (3.2)
Living situation	Living with spouse and children	182 (47.9)
	Living with spouse	112 (29.4)
	Living with children	50 (13.2)
	Living alone	36 (9.5)

Prior to the analysis, the assumptions of SEM including normal distribution, error independence, and multiple alignment were examined. To examine the normal distribution of the research variables, the skewness and kurtosis of the distribution of scores were used, the results of which showed that the distribution of scores of all variables is normal (range of distribution between +2 and -2). The Dorbin-Watson test was used to check the independence of the errors, the results of which showed no correlation between the errors (D.W=1.84, range between 1.5 and 2.5 is acceptable). Inflation variance (VIF) and tolerance factors were used to evaluate the multiple alignment between the predictor variables. The results showed that there is no alignment between the variables (VIF amplitude was less than 10 and tolerance was higher than 0.1). Another assumption is the establishment of a linear relationship between independent and dependent variables, which was examined by Pearson’s correlation, the results of which are reported along with the mean and standard deviation of the study variables in Table 2.

**Table 2:** The mean, standard deviation, and correlation of the variables

variable	M	SD	1	2	3	4	5
Avoidant attachment style	9.31	2.48	-				
Secure attachment style	13.97	2.50	-0.35^**^	-			
Ambivalent attachment style	10.66	2.93	0.29^**^	-0.17^**^	-		
PR	52.79	8.25	-0.26^**^	0.48^**^	-0.24^**^	-	
NR	44.32	9.06	0.35^**^	-0.32^**^	0.46^**^	-0.05	-
Happiness	67.62	15.12	-0.38^**^	0.39^**^	-0.32^**^	0.47^**^	-0.43^**^

^*^P< 0.05, **P< 0.01

**Fig. 2:**
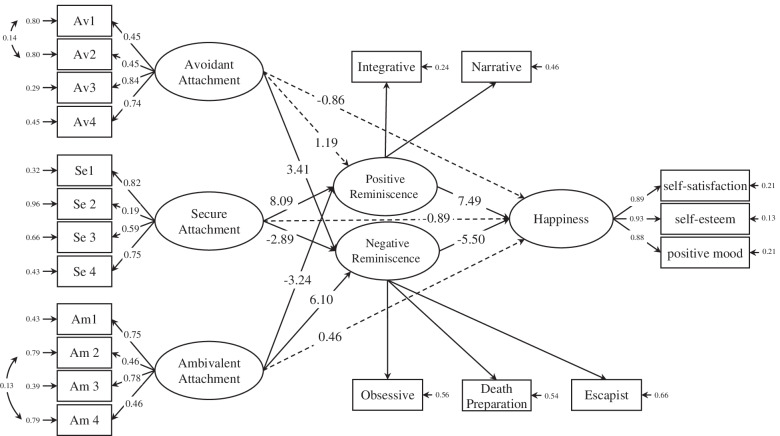
The structural model for the relationship between attachment styles and happiness in the mediating role of reminiscence styles in the elderly

As can be seen in Table 2, PR is correlated significantly with avoidance attachment style (*r*=-0.26, *p*<0.01), secure attachment style (*r*=0.48, *p*<0.01) and ambivalent attachment style (*r*=0.24, *p*<0.01). In addition, NR is correlated significantly with avoidance attachment style (*r*=0.35, *p*<0.01), secure attachment style (*r*=-0.32, *p*<0.01) and ambivalent attachment style (*r*=0.46, *p*<0.01). Moreover, happiness is associated significantly with PR (*r*=0.47, *p*<0.01) and NR (*r*=-0.43, *p*<0.01). There is also a significant relationship between happiness with avoidance attachment style (*r*=-0.38, *p*<0.01), secure attachment style (*r*=0.39, *p*<0.01) and ambivalent attachment style (*r*=-0.32, *p*<0.01). Utilized to test the research hypotheses was model fit. Accordingly, the structural equation model was used in order to evaluate the hypothesized model of the study. Shown in the Fig. 2 is the final structural model for the relationship between attachment styles and happiness through the mediating role of reminiscence styles.

First, considered to determine the overall fit of the model was the fit index. The model fit indices are presented in Table 3. For the X^2^/df fit index, values ​​smaller than 3 are appropriate, and the closer it is to zero, the better the model will fit. For GFI, IFI, CFI and NFI indices, a value close to 0.90 and above is considered as an acceptable goodness of fit, which indicates that the model is good. In relation to the RMSEA index, values ​​close to 0.05 or less indicate a good fit of the model and a value of 0.08 or less indicates a reasonable error of approximation. A value higher than 0.10 indicates the need to reject the model [42]. The fit indices presented in Table 3 indicate the good fit of the model.

**Table 3:** Fit indices for the developed model

Model fit indices	X^2^	df	X^2^/ df	GFI	IFI	NFI	CFI	RMSEA
Obtained values	424.68	154	2.75	0.9	0.95	0.92	0.95	0.068

Then, all the effects related to different paths in the SEM were considered, the results of which are presented in Table 4.

**Table 4:** Coefficients of the model of the relationship between MS an attachment styles through SE and NM

Direct path	Regression coefficient	t
Avoidant → PR	0.08	1.19
Avoidant → NR	0.25	3.41**
Avoidant → Happiness	-0.05	-0.86
Secure → PR	0.55	8.09**
Secure → NR	-0.20	-2.89**
Secure → Happiness	-0.06	-0.89
Ambivalent → PR	-0.20	-3.24**
Ambivalent → NR	0.43	6.1**
Ambivalent → Happiness	0.03	0.46
PR → Happiness	0.54	7.49**
NR → Happiness	-0.47	-5.5**

^**^P< 0.01

*NR* Negative reminiscence, *PR* Positive reminiscence

Analysis of data obtained from standard coefficients in the SEM presented in Table 4 shows that the direct effect of avoidance attachment style on NR (β=0.25, *p*<0.01) was positive and significant. However, the effect of avoidance attachment style on PR (β=0.08 *p*>0.05) and happiness (β=0.08, *p*>0.05) was not significant. Although secure attachment style had a positive and negative significant direct effect on PR (β=0.55, *p*<0.01) and NR (β=-0.20, *p*<0.01), respectively, it did not have any significant direct effect on happiness (β=0.06, *p*>0.05). Moreover, ambivalent reminiscence style had a positive and negative significant direct effect on PR (β=-0.20, *p*<0.01) and NR (β=0.43, *p*<0.01), respectively. However, the direct effect of ambivalent attachment style on happiness was not significant (β=0.03, *p*>0.05). PR showed a direct significant positive effect on happiness (β=0.54, *p*<0.01), and NR showed a direct significant negative effect on happiness (β=-0.47, *p*<0.01). The Sobel’s test was also used to investigate the mediating role of reminiscence styles in the relationship between attachment styles and happiness, the results of which are reported in Table 5.

**Table 5:** results of Sobel’s test for investigating the mediating role of PR and NR in the relationship between attachment styles and happiness

variables	Indirect coefficient	Sobel’s test (z)	P
Avoidant → PR → Happiness	0.04	-1.87	0.06
Avoidant → NR → Happiness	-0.12	-6.01	0.001
Secure → PR → Happiness	0.29	7.85	0.001
Secure → NR → Happiness	0.09	5.66	0.001
Ambivalent → PR → Happiness	-0.10	-4.38	0.001
Ambivalent → NR → Happiness	-0.20	-7.22	0.001

*NR* Negative reminiscence, *PR* Positive reminiscence

Presented in Table 5, the results of Sobel’s test indicated that PR does not serve a significant mediating role in the relationship between avoidance reminiscence style and happiness (Z=-1.87, *p*>0.05). NR, however, played a significant mediating role in the relationship between avoidance reminiscence style and happiness (Z=6.01, *p*<0.01). In addition, both PR (Z=7.85, *p*<.01) and NR (Z=5.66, *p*<0.01) had a significant mediating effect in the relationship between secure attachment style and happiness. Finally, both PR (Z=-4.38, *p*<0.01) and NR (Z=7.22, *p*<0.01) indicated a significant mediator role in the relationship between ambivalent attachment style and happiness.

In order to test the seventh and eighth hypotheses of the study, considered in the first step was the role of individuals’ gender in the extent of reminiscence. Results showed that the mean score of integrative (*t*_(378)_ =-3.472, *p*=0.001), narrative-transmissive (*t*_(378)_ =-3.470, *p*=0.001) and positive (*t*_(378)_ =-3.826, *p*<.001) reminiscences are higher in men than in women. Instead, women had higher mean score of obsessive reminiscence (*t*_(378)_ =4.332, *p*<0.001) than men. There was no significant gender difference in the other styles of reminiscence. In addition, Pearson’s correlation coefficient was applied to examine the relationship between age (ranged from 60 to 95 years) and reminiscence styles. Results demonstrated that participants’ age is positively correlated with narrative-transmissive (*r*_(378)_= 0.213, *p*<0.001), obsessive (r _(378)_ =0.138 , *p*=0.007), escapist (r _(378)_=0.162, *p*=0.002), death preparation (r _(378)_ =0.215, *p*<0.001), positive (r _(378)_ =0.143, *p*=0.005), and negative (r _(378)_=0.222, *p*<0.001) reminiscence styles. The same relationship, however, did not hold for integrative reminiscence (r _(378)_ =0.022, *p*=0.672). In the second step, the moderating effect of age and gender on the relationship between PR and NR with happiness was examined using hierarchical regression. As such, first, the effect of age and gender were entered into the model, and then, the reminiscence styles, and finally, the interactive effect of gender and age with reminiscence styles were entered into the model. Presented in Table 6, results indicated that the effect of gender, age and both styles of reminiscence are significant in predicting happiness, but none of the interactive effects is significant predictor of happiness. In other words, the age and gender of the participants did not have a significant effect on the relationship between reminiscence and happiness. A similar model was performed to investigate the moderating effect of age and gender on the relationship between attachment styles and reminiscence styles. Shown in the Table 6, results displayed no significant effect. In other words, the age and gender of the participants did not have a significant effect on the relationship between attachment styles and reminiscence styles.

**Table 6:** Summary of hierarchical regression analysis for positive and NR and their interactions with gender and gender in predicting happiness in the elderly

predictors	R^2^	B	SEM	Beta	t
PR					
Model 1: Age	0.033	-0.312	0.104	-0.152	-2.993^**^
Gender		4.259	1.499	0.145	2.842^**^
Model 2: Age	0.167	0.676	0.086	0.378	-4.160^***^
Gender		-0.405	0.097	-0.198	1.614
PR		2.281	1.414	0.077	7.846^***^
Model 3: Age	0.174	-1.543	0.661	-0.754	-2.336^*^
Gender		-7.191	9.532	-0.244	-0.754
PR		-0.787	0.801	-0.440	-0.983
Gender × PR		0.021	0.012	1.020	1.724
Age × PR		0.176	0.178	0.332	0.991
NR					
Model 1: Age	0.033	-0.312	0.104	-0.152	-2.993^**^
Gender		4.259	1.499	0.145	2.842^**^
Model 2: Age	0.069	-0.214	0.105	-0.105	-2.033^*^
Gender		3.554	1.481	0.121	2.399^*^
NR		-0.341	0.086	-0.202	-3.944^***^
Model 3: Age	0.068	0.303	0.570	0.148	0.531
Gender		-3.538	7.777	-0.120	-0.455
NR		0.332	0.799	0.196	0.415
Gender × NR		-0.011	0.012	-0.931	-0.931
Age × NR		0.158	0.170	0.930	0.930

^*^P<.05, ^**^P<.01, ^***^P<.001

*NR* Negative reminiscence, *PR* Positive reminiscence

## Discussion

The current study aims to investigate the relationship between attachment styles and happiness through the mediating role of reminiscence styles in the elderly. The results of SEM revealed that the hypothesized model of this study has a good fit in the study sample. As such, secure and ambivalent attachment styles were significantly associated with happiness through both PR and NR, and avoidant attachment style was significantly associated with happiness only through NR. Moreover, participants’ age and gender did not have a significant moderating effect on the relationship between attachment styles and reminiscence styles, as well as on the relationship between reminiscence styles and happiness. In conclusion, the second to sixth hypotheses of the study were confirmed and the first, seventh and eighth ones were not confirmed.

The findings of the current study regarding avoidant and secure individuals are in line with that of Webster [16], revealing that avoidant and secure people have lower extent of NR compared to preoccupied and fearful ones. The current study also found that the degree of correlation between secure and avoidance attachment styles with NR are lower than degree of correlation between ambivalent attachment style and NR. In addition, as with Webster’s [16], the findings of the current study indicated that secure attachment style has a significant positive relationship with PR. The mentioned relationship was negative for ambivalent attachment style, and was not significant for avoidant attachment style. The findings of the present study are somewhat consistent with that of Dunlop [49], which showed that avoidant attachment style has a significant negative relationship with the storytelling enjoyment and narrative mindset in adults. Likewise, the finding of this study regarding the moderating effect of gender on the relationship between reminiscence and happiness was consistent with that of Webster [16] and inconsistent with that of Webster and McCall [41]. The finding of this study regarding the moderating effect of age on the relationship between reminiscence and happiness was inconsistent with that of Webster and McCall [41].

### Attachment styles and reminiscence

The central role of reminiscence and the content of some kind of memories, in the attachment theory, has itself drown from internal working models (IWMs). Accordingly, Bowlby [50] stated that IWMs, emerged from the daily child-parent interactions [51], are relatively stable structures that persist in adolescence and adulthood and shape individuals’ behaviors. The basic aspects of IWMs embed in individuals’ various types of memories (e.g., procedural, sensory, semantic, episodic, connotative, and working). Among mentioned types of memories, episodic and autobiographical ones play an important role in the function of individuals’ IWMs [52]. These memories, which integrate cognitions and emotions by gathering information from different parts of the brain, play a significant role in people’s ability to tell stories or to reminisce [52]. Consequently, the presence of different kind of IWMs in avoidant, ambivalent, and secure individuals are closely related to their ability of reminiscing. In a way that, avoidant people usually have narratives that are based on shutting down episodic memory and removing emotions, leading to dry and formal narratives. Some of the other essential features of avoidant people’s narratives are the minimization of negative experiences, and the strong denial of negative emotions. Ambivalent people, often have confusing narratives that are difficult to follow. They give a lot of information about their emotions. Coming from different types of memory such as semantic and episodic ones, this information is often contradictory and does not have an accurate timing. Other characteristics of ambivalent narratives include the presence of irrelevant details during the discussion, passive semantic thoughts that usually do not lead to a specific conclusion or point, and the stream of consciousness during speech, without a focused and clear direction. At last, secure people have a fine coherence between their memory systems and have a well capability to remember positive and negative events, to react to them, and to integrate them. Some of the other characteristics of the narrations of secure people are limited speech dysfluency, not to the extent of distorting information, simultaneous recognition of contradictions and new thoughts, lack of emotional disturbance at the time of narration, clarity of time and place in narrations, even during disruptive narration [52].

### Reminiscence styles and happiness

Bryant et al. [40] believe that there are several factors that contribute to happiness in the elderly who engage in PR and avoid dealing with NR. They believe that creating insights into the self and the present time, as well as mental imagery of the old times are two factors that create positive emotions and happiness during reminiscence. They explicitly state that the adaptive value of reminiscence is not in escaping the problems of the present time and taking refuge in the past times, but rather reminiscing is a constructive way for creating a sense of insight into the present time. The elderly at this age are looking to reintegrate different components of their lives to do their final developmental task. In fact, reminiscence helps them put these components together again and gain some kind of insight into themselves and their age. Therefore, they may experience positive emotions through reminiscing about past times, and as a result, feel happier. Moreover, the mental imagery of past positive events in the elderly may remind them of the same positive emotions. Bryant et al. [40] believe that in mental imagery, what creates positive emotions in a person is the imagination about memories, and the mental imagery increases this imagination about past memories. During mental imagery, the details of some particular memories, which can sometimes be negative, are not remembered, in turn may lead the elderly to feel happier.

### Attachment styles, reminiscence styles and happiness

Based on the previous two sections, it can be concluded that secure people, on the one hand, had caregivers who interacted a lot with their children and promoted their children’s narration and reminiscence. Having both a better memory capability and a greater ability to reminisce their memories, along with greater openness and comfort in interpersonal communication, secure people are more likely to tend to express memories in the form of coherent and attractive narratives with an accurate structure. On the other hand, being natured by parents who provide a safe haven for them and a secure base for their exploration of the world around them, secure people have IWMs formed based on the security of the world around them. Therefore, they explore the world around them more confidently, which in turn provide them with the opportunity in old age to transmit their experiences in the form of reminiscence about their memories to other people and the next generations. Thus, high extent of PR in secure people cause them to experience more positive emotions and feel happier. In addition, because of their high ability to integrate their cognition and emotions, they are less likely to engage in maladaptive strategies such as NR to escape current negative emotions and move toward death.

People with avoidant attachment style, however, tend to shut down episodic and autobiographical memories due to their IWMs formed based on the low level of interaction and dry and soulless atmosphere between them and their caregivers. Due to lack of interpersonal skills and unwillingness to establish a relationship, avoidant people have neither much ability to reminisce in form of narratives and stories, nor much interaction to reminisce their memories in the context of those interactions. Further, it seems that their desire to minimize negative experiences and deny negative emotions leads them to apply escapist and death preparation reminiscences, which in turn can cause them to experience negative emotions and lower level of happiness.

Finally, ambivalent individuals whose caregivers provided them with an environment full of contradictions and contradictory messages may not be able to form suitable narratives and reminiscences. They also are not capable to present coherent and integrated reminiscences with a clear message, and their messages during the reminiscence are sometimes contradictory, without accurate timing. This can lead to their reluctance to express narrative-transmissive reminiscence and, as a result, reduces their sense of happiness.

### The limitations of the study

One of the limitations of this study is date collection through self-assessment questionnaires. Although these questionnaires provide useful information, they can sometimes reduce the validity of the results themselves. Meanwhile, although SEM is used in this study, still the nature of the obtained relationships is of the relational type and not causal, and due to the statistical method used and the cross-sectional nature of this study, causal perceptions are not suitable for this type of study. In addition, this study has been used a sample of Iranians, and due to the deep cultural differences in Eastern and Western societies, extreme caution should be exercised in extending these findings to other societies. It should be noted, however, that theories of an ecological nature, such as the attachment theory, are relevant to the human species, and that cultural divisions are less influential than in other areas.

### Suggestions for further studies

Understanding the role of attachment styles in the formation of the reminiscence styles, and reviewing this process from childhood to adulthood and old age, along with explaining how it relates to happiness, will provide therapists working in the field of the elderly with an in-depth look at this issue. Emphasizing the interaction of parents with their children in the formation of IWMs and its survival into old age and its role in the reminiscence styles of individuals, this article provides some of the necessary elements for building reminiscence therapy protocols for working with the elderly. It is suggested that future studies use highly precisionist tools and control disturbing variables to examine the relationship expressed in this study. These studies can use adult attachment interviews to determine individuals’ attachment style, and coding people’s reminiscence to examine their reminiscence styles, and use longitudinal designs to provide a causal relationship between these psychological structures. Besides, re-conducting this type of study in different cultural contexts helps to form more accurate and universal findings in this field.

## Data Availability

The datasets generated and/or analysed during the current study are not publicly available due to some legal limitations imposed by ethics committee of Kermanshah University of Medical Sciences, Iran, but are available from the corresponding author on reasonable request.
